# Dissecting Integrin Expression and Function on Memory B Cells in Mice and Humans in Autoimmunity

**DOI:** 10.3389/fimmu.2019.00534

**Published:** 2019-03-21

**Authors:** Alessandro Camponeschi, Natalija Gerasimcik, Ying Wang, Timothy Fredriksson, Dongfeng Chen, Chiara Farroni, Katrin Thorarinsdottir, Louise Sjökvist Ottsjö, Alaitz Aranburu, Susanna Cardell, Rita Carsetti, Inger Gjertsson, Inga-Lill Mårtensson, Ola Grimsholm

**Affiliations:** ^1^Department of Rheumatology and Inflammation Research, University of Gothenburg, Gothenburg, Sweden; ^2^Department of Microbiology and Immunology, University of Gothenburg, Gothenburg, Sweden; ^3^Institute of Life Sciences, Jiangsu University, Zhenjiang, China; ^4^B Cell Physiopathology Unit, Immunology Research Area, Bambino Gesù Children's Hospital IRCSS, Rome, Italy; ^5^Unit of Diagnostic Immunology, Department of Laboratories, Bambino Gesù Children's Hospital IRCCS, Rome, Italy

**Keywords:** integrins, LFA-1, VLA-4, memory B cells, adhesion, spleen, autoimmunity

## Abstract

Immunological memory ensures life-long protection against previously encountered pathogens, and in mice and humans the spleen is an important reservoir for long-lived memory B cells (MBCs). It is well-established that integrins play several crucial roles in lymphocyte survival and trafficking, but their involvement in the retention of MBCs in secondary lymphoid organs, and differences between B cell subsets in their adhesion capacity to ICAM-1 and/or VCAM-1 have not yet been confirmed. Here, we use an autoimmune mouse model, where MBCs are abundant, to show that the highest levels of LFA-1 and VLA-4 amongst B cells are found on MBCs. *In vivo* blockade of VLA-4 alone or in combination with LFA-1, but not LFA-1 alone, causes a release of MBCs from the spleen into the blood stream. In humans, we find that in peripheral blood, spleens, and tonsils from healthy donors the highest expression levels of the integrins LFA-1 and VLA-4 are also found on MBCs. Consistent with this, we found MBCs to have a higher capacity to adhere to ICAM-1 and VCAM-1 than naïve B cells. In patients with the autoimmune disease rheumatoid arthritis, it is the MBCs that have the highest levels of LFA-1 and VLA-4; moreover, compared with healthy donors, naïve B and MBCs of patients receiving anti-TNF medication have enhanced levels of the active form of LFA-1. Commensurate levels of the active αL subunit can be induced on B cells from healthy donors by exposure to the integrin ligands. Thus, our findings establish the selective use of the integrins LFA-1 and VLA-4 in the localization and adhesion of MBCs in both mice and humans.

## Introduction

Interactions between integrins and their ligands are essential for maintaining the location of leukocytes in general, and mediate the extravasation of cells into tissues as well as their retention in peripheral lymphoid organs (1). In particular, marginal zone (MZ) B cells not only express higher levels of the LFA-1 (αLβ2) and VLA-4 (α4β1) integrins than do their neighbors the follicular (FO) B cells (2), but also depend on these receptors for their retention in the splenic marginal zone; MZ B cells retained in the spleen are released to the circulation when the interaction with ICAM-1 and VCAM-1 is blocked, both in wild-type and in autoimmune mice (2, 3). Appropriately, the LFA-1 and VLA-4 ligands, ICAM-1 and VCAM-1, are expressed in the splenic marginal sinus in mice (2), and VCAM-1 is expressed in the perifollicular and marginal zone in the human spleen (4).

Memory B cells (MBCs) are key components of immunological memory, and consequently serve as mediators of disease when they react to self-antigens in autoimmunity. In humans, unswitched MBCs (also referred to as IgM MBCs or MZ-like B cells) express high levels of the LFA-1 subunit β2 (5), and the VLA-4 integrin is involved in the activation of MBCs (6). As well as harboring MZ B cells, the spleen is an important reservoir for MBCs (7–9); although to date the mechanism of their retention there has not been identified, LFA-1 and/or VLA-4 integrins are obvious candidates for that role. Here we investigate this possibility using SLC^−/−^ (surrogate light chain-deficient) mice, which share features with autoimmune mouse models, including spontaneous formation of germinal centers (GCs) and MBCs (3, 10–12). Furthermore, in humans we examine the expression pattern of the LFA-1 and VLA-4 integrins in B-cell subsets from secondary lymphoid organs as well as functional properties of these integrins in PB of healthy donors and patients with rheumatoid arthritis.

## Materials and Methods

### Mice

SLC^−/−^ mice (13), previously backcrossed for >10 generations on the C57BL6/OlaHsd background, which lacks alpha-synuclein (14), were bred after embryo transfer onto the C57BL6/NCrl background, and intercrossed to establish SLC^−/−^ mice with an intact alpha-synuclein locus. Mice were kept at the University of Gothenburg (EBM) SPF animal facility and bred under project license authorization (2013/81 and 2016/10). Control mice (C57Bl/6NCrl) were purchased from Charles River (Germany) and Taconic (Denmark). Female mice aged 5–6 months were used throughout.

### Human Samples

Peripheral blood mononuclear cells (PBMCs), tonsils and spleens were used for the staining of integrins. The spleens were from children (6–10 years of age) undergoing surgery due to vessel malformations. The study on the splenic samples was approved by the Ethical Committee of Ospedale Pediatrico Bambino Gesù in Rome. Informed consent was obtained from the children's parents. Tonsils were collected from children (1–9 years of age) undergoing tonsillotomy. As no personal information or identity was recorded, no written consent or approval by the Human Research Ethics Committee was needed (Swedish law 2003: 460, paragraphs 4 and 13). PBMCs from patients with RA (57 ± 12 years of age) positive for both rheumatoid factor and antibodies to citrullinated proteins, except two patients that were positive only for rheumatoid factor (Supplementary Table 1), were obtained from the Sahlgrenska University Hospital (660-11, approved 2011-09-07; T996-13, approved 2013-12-12 and 334-15, approved 2015-06-11 all by the Ethical Committee of Gothenburg). In terms of the controls for PBMCs, as we did not have sufficient numbers of age- and sex-matched controls we used additional controls that were not matched. Comparing the two control groups used in our study, showed no significant differences in integrin expression. The study was performed following the guidelines of the Declaration of Helsinki.

### Flow Cytometry

Single cell suspensions were stained with a cocktail of antibodies using a PBS buffer containing 3% (mouse) or 10% (human) FCS and 1 mM EDTA (see Supplementary Tables 2, 3 for a complete list of Abs recognizing mouse and human antigens) following standard techniques, and analyzed on LSRII^TM^, FACSVerse^TM^, FACSLyric^TM^, or LSRFortessa^TM^ X-20 (all BD Biosciences) flow cytometers. Data were analyzed using FlowJo software version 10 (Treestar Inc.).

### Treatment With Antibodies Recognizing α4 and LFA-1 Integrins

This treatment has been described previously (2, 3). Briefly, SLC^−/−^ mice were injected on days 0, or 0 and 5, i.p. with anti-α4 (clone: M17/4; 100 μg) and/or anti-LFA-1 (clone: PS/2; 100 μg) or isotype control antibodies (BioXcell, USA), and sacrificed after 5 h or at day 14. At the time of sacrifice, blood, and spleens were collected.

### Adhesion Assay

CD19^+^ B cells were purified from PBMCs by negative immunomagnetic sorting (Dynabeads® Untouched™ Human B Cells Kit, Invitrogen, Life Technologies). After washing, cells were resuspended in RPMI with 10% FCS at a concentration of 5 x 10^5^ cells/ml. High absorbance plates (Immulon 4 HBX, Extra High Binding, 96 wells, Flat-bottom, VWR) were coated with the ligands (VCAM-1 5 μg/ml; ICAM-1 10 μg/ml) at 37°C for 2 h in 50 μl carbonate buffer. The plate was decanted and then incubated with 200 μl PBS with 1% BSA (1 g BSA in 100 ml PBS) for 1 h at 37°C to block non-specific adhesion. The plate was washed once with RPMI before use. 5 x 10^4^ cells (in 100 μl RPMI with 10% FCS) were added to the wells and incubated for 30 min at 37°C. Non-adherent cells were removed by gentle washing. Subsequently, adherent cells were removed by incubating for 15 min on ice in RPMI with 5 mM EDTA. Both adherent and non-adherent cells were washed once and then stained for flow cytometry.

### Pre-treatment of PBMCs With ICAM-1 and VCAM-1 and Staining for Active Conformation of αL Integrin Subunit

PBMCs from HDs and patients with RA under anti-TNF medication were pre-treated with ICAM-1 (20 μg/ml) and VCAM-1 (10 μg/ml) at 37°C for 1 h in RPMI with 10% FCS. Thereafter the cells were stained for the active form of αL with the standard panel of antibodies (Supplementary Table 3).

### Statistics

Outliers were removed by using the ROUT method with Q set to 1%. Shapiro-Wilk test was used to evaluate normality. The statistical significance of differences between tissues and treatments was determined using tests as appropriate: an unpaired or paired two-tailed *t*-test or ordinary one-way ANOVA with Tukey's multiple comparisons, using Graph-Pad Prism version 7 (La Jolla, CA, USA). Significance levels are represented on figures by asterisks: ^*^p < 0.05; ^**^p < 0.01; ^***^p < 0.001; ^****^p < 0.0001.

## Results

### Sustained Treatment With Anti-integrin Antibodies Depletes MBCs in the Spleen

**Figure 1:**
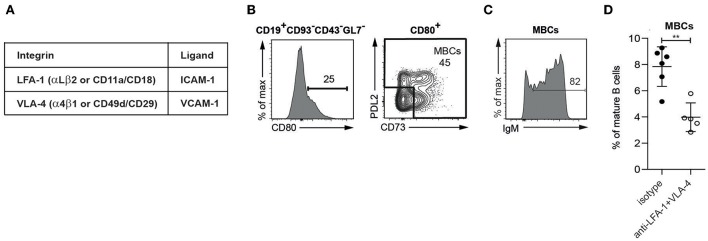
MBCs present in the spleen of SLC^−/−^ mice are dependent on integrins for their retention **(A)**. The integrins (subunits) of interest and their ligands **(B–D)**. Flow cytometric analysis of spleen from SLC^−/−^ mice **(B)** Gating strategy for MBCs **(C)** Percentage of IgM-expressing cells in MBCs **(D)** Percentages of MBCs isolated from spleens of SLC^−/−^ mice treated for 2 weeks with anti-LFA-1 and anti-α4. *n* = 6 (treated), *n* = 5 (isotype control); error bars show mean +/–SD; data are representative of two independent experiments. An unpaired two-tailed Student *t-*test was used (^**^p < 0.01).

The integrins of interest in this study are LFA-1 and VLA-4, and their ligands ICAM-1 and VCAM-1 (Figure 1A). Starting with a population of mature B cells identified as CD19^+^CD93^−^CD43^−^GL7^−^ lymphocytes, MBCs were defined as CD80^+^CD73^+/−^PDL2^+/−^ based on the differential expression of the CD80, CD73, and PDL2 surface markers (15), (Supplementary Figure 1; Figure 1B). These are to be described in more detail elsewhere (Aranburu et. al. in preparation); here it suffices to note that the MBCs in SLC^−/−^ mice contain mainly IgM-expressing cells (Figure 1C).

To determine whether the adhesion of mouse MBCs in the spleen depends on integrins, we treated SLC^−/−^ mice with antibodies against LFA-1 and VLA-4. After a 2-week period, the presence of MBCs was significantly reduced (Figure 1D), showing that MBCs rely on the interaction with ICAM-1 and VCAM-1 for their retention in the spleen.

### Acute Treatment With anti-VLA-4 Antibodies Induces the Release of MBCs Into PB

**Figure 2:**
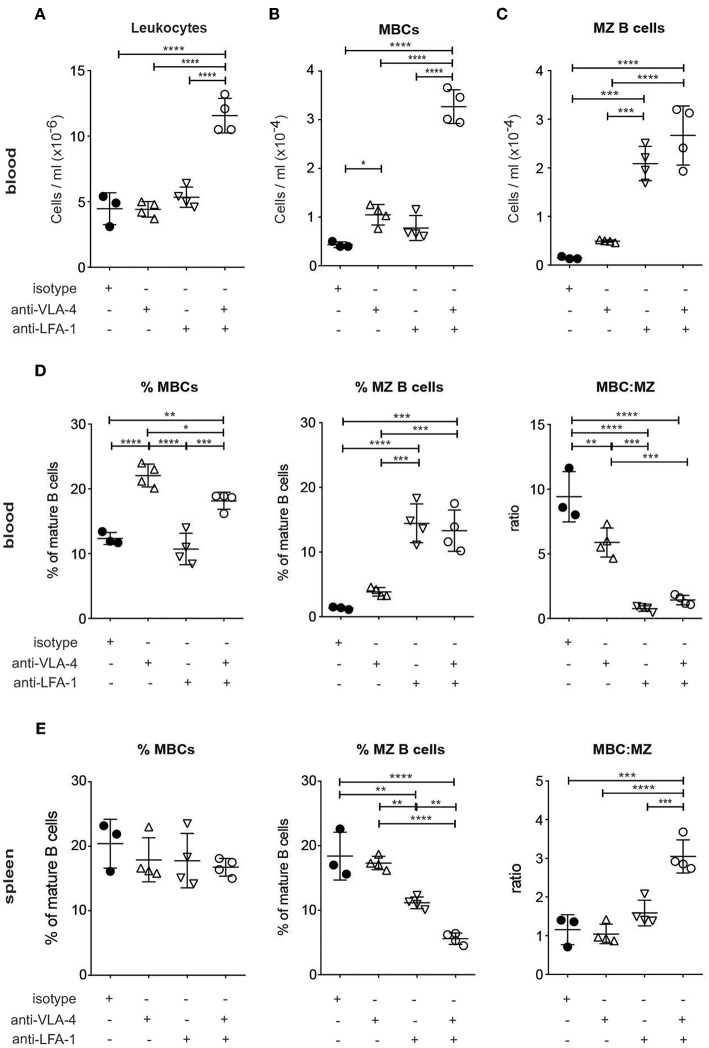
MBCs are acutely dependent on VLA-4 for their retention in the spleen with an additive requirement of LFA-1 **(A–D)**. Flow cytometric analysis of PB taken from SLC^−/−^ mice after 5 h treatment with anti-LFA-1 and/or anti-α4 antibodies **(A)** Numbers of leukocytes **(B)** Numbers of MBCs **(C)** Numbers of MZ B cells **(D)** %MBCs as proportions of mature B cells (left panel), % of MZ B cells as proportions of mature B cells (middle panel) and ratio of MBC:MZ expressed as proportions of mature B cells (right panel) **(E)**. Flow cytometric analysis of spleen cells: %MBCs as proportions of mature B cells (left panel), % of MZ B cells as proportions of mature B cells (middle panel) and ratio of MBC:MZ expressed as proportions of mature B cells (right panel). *n* = 4 (treated) and *n* = 3 (isotype control); error bars show mean +/–SD; data are representative of two independent experiments. One-way ANOVA with Tukey's multiple comparisons was used (^*^p < 0.05, ^**^p < 0.01, ^***^p < 0.001, ^****^p < 0.0001).

To investigate whether the observed integrin-mediated loss of MBCs from the spleen resulted in their accumulation in the circulation, we started by looking at the number of leukocytes in the PB of SLC^−/−^ mice soon (5 h) after the injection of the blocking antibodies. Compared to the injection of control antibodies, leukocyte number more than doubled after the injection of antibodies against both LFA-1 and VLA-4 together, but did not alter significantly when each antibody was used alone (Figure 2A). This is to be contrasted with the situation for the MBCs, where the anti-VLA-4 acted selectively, increasing their release into the blood (Supplementary Figure 2; Figure 2B). On the other hand, the number of MZ B cells was selectively increased by anti-LFA-1 treatment, and blocking with both antibodies increased numbers of MBCs as well as MZ B cells at least 3-fold (Figures 2B,C). Comparison of the proportions of MBCs and MZ B cells as well as their ratios in the blood (Figure 2D) shows the selectivity of anti-VLA-4 treatment for MBC release, whereas MZ B cells release was more dependent on anti-LFA-1 and double-blocking. Flow cytometric analysis of spleen B cell populations 5 h after antibody treatment confirmed the loss of MZ B cells relative to MBCs due to treatment with both antibodies (Figure 2E). These data show that the two integrins of interest have additive effects on the retention of both MZ B cells and MBCs, but that the selectively important partners are LFA-1 for MZ B cells and VLA-4 for MBCs.

### Elevated Integrin Expression on MBCs in SLC^−/−^ Mice

**Figure 3:**
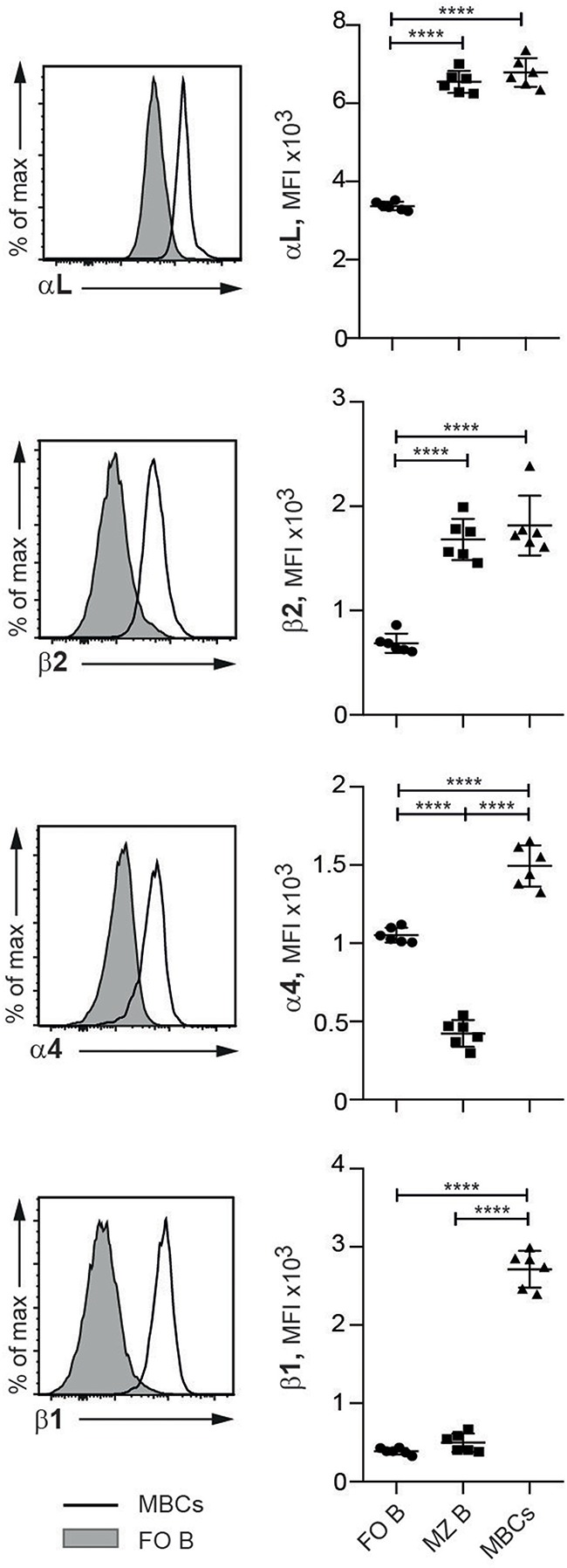
MBCs express higher levels of LFA-1 and VLA-4 than FO and MZ B cells in SLC^−/−^ mice. Flow cytometric analysis of spleen cells from SLC^−/−^ mice. Histograms show the expression of integrins on FO B and MBCs and graphs show the MFIs of integrin subunits αL, β2, α4, and β1 expressed on FO B, MZ B and MBCs. *n* = 6, data are from three independent experiments; error bars show mean +/–SD. One-way ANOVA with Tukey's multiple comparisons was used (^****^p < 0.0001).

Given our observations on their functional effects, we set out to determine the appearance of the four integrin subunits studied with respect to the various mature B cell subsets in SLC^−/−^ mice. Compared to FO B cells, MZ B cells and MBCs expressed at least twice the levels of the LFA-1 integrins (Figure 3). For the VLA-4 subunits, however, it was the MZ B cell subset that expressed the lowest levels, and MBCs by comparison demonstrated several-fold higher expression. These data sit well with our observations on the selective release of MBCs and MZ B cells into the PB, and single out the MBCs as distinct from the naïve B cells (FO and MZ) in expressing high levels of all four integrin subunits, a feature that is likely to be pertinent to the autoimmune status of the SLC^−/−^ mice.

### Integrin Expression Is Elevated on Human MBCs in Peripheral Blood and Secondary Lymphoid Organs

**Figure 4:**
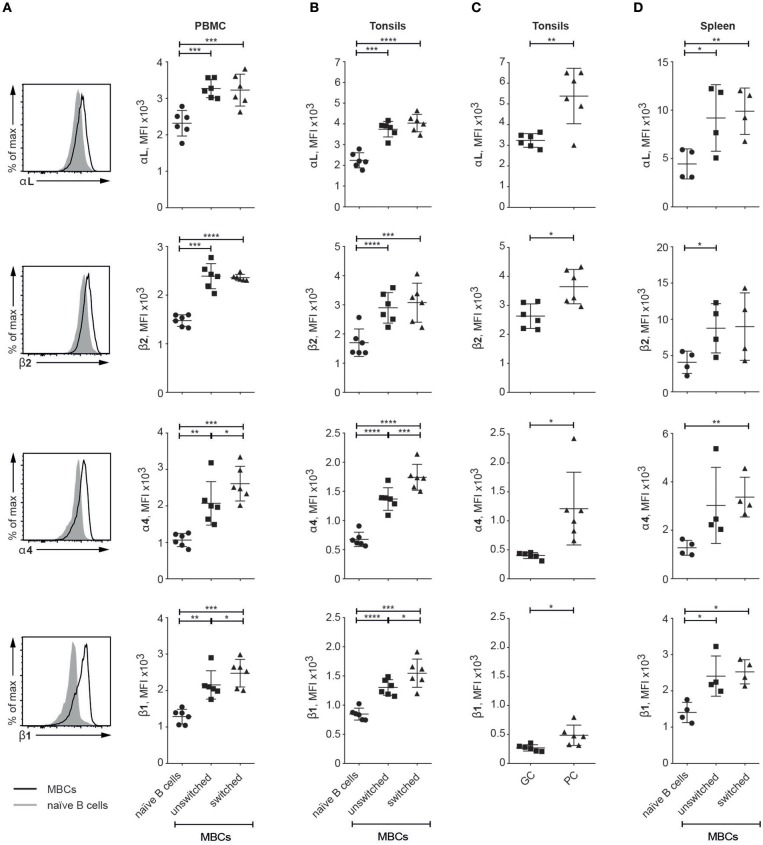
Human MBCs express higher levels of the integrins of interest than naïve B cells **(A–D)**. Flow cytometric analysis of human tissues; graphs compare naïve B cells with unswitched and switched MBC populations as well as GC B cells and plasma cells (PCs) (in **C**) for their levels of expression of the αL, β2, α4, and β1 integrin subunits **(A)** PBMC Histograms compare integrin expression levels between naïve B and all MBCs **(B)** Tonsil **(C)** Tonsil GC B cell and PCs **(D)** Spleen. Data are from at least two independent experiments; error bars show mean +/– SD. An unpaired two-tailed Student *t-*test was used (^*^p < 0.05, ^**^p < 0.01, ^***^p < 0.001, ^****^p < 0.0001).

Looking for relevance of our data from SLC^−/−^ mice to human immunity, we investigated the expression patterns of the integrin subunits of interest in human tissues: PBMC, tonsil and spleen (for gating strategy see Supplementary Figure 3). In all three tissues, MBCs (unswitched and switched) expressed higher levels of all four integrin subunits of interest than did their naïve counterparts; these elevations were statistically significant in all cases except for β2 (naïve vs. switched MBCs) and α4 (naïve vs. unswitched MBC) in the spleen (Figures 4A,B,D). Further, both VLA-4 subunits were significantly more highly expressed on switched than on unswitched MBCs from PBMCs and tonsils (Figures 4A,B). Gating on GC B and plasma cells in the tonsils, we also noticed that the levels of all four integrin subunits were higher on the latter (Figure 4C). Thus, in humans as in mice, the integrins of interest here are expressed at higher levels on MBCs than on naïve B cells.

### Greater Adhesion Capacity to ICAM-1 and VCAM-1 in Human MBCs Than in Naïve B Cells

**Figure 5:**
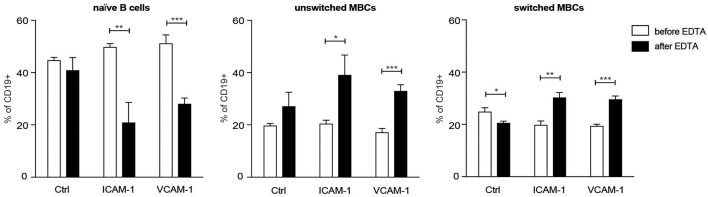
Human unswitched and switched MBCs show a higher adhesion capacity to ICAM-1 and VCAM-1 than do naïve B cells. Flow cytometric analysis of CD19^+^ B cells from PB of HDs tested for adhesion to the integrins of interest. Shown are the proportions of naïve B cells, unswitched MBCs, and switched MBCs harvested before and after treatment with EDTA. *n* = 3; data are representative of two independent experiments. An unpaired two-tailed Student *t-*test was used (^*^p < 0.05, ^**^p < 0.01, ^***^p < 0.001).

To determine whether the enhanced expression of LFA-1 and VLA-4 integrins on human MBCs compared to naïve B cells was related to adhesion capacity, we performed an *ex vivo* adhesion assay (2) on human B cell subsets. A higher proportion of the naïve B cells were found in the non-adherent fraction (before EDTA) whereas the unswitched and switched MBCs showed the opposite pattern (Figure 5), thus demonstrating a higher adhesion capacity to ICAM-1 and VCAM-1 than the naïve B cells. The *ex vivo* demonstration of MBC adhesion to the integrin ligands is complementary support of the *in vivo* data from our mouse model.

### Higher Than Normal LFA-1 Expression on MBCs in Patients With RA

**Figure 6:**
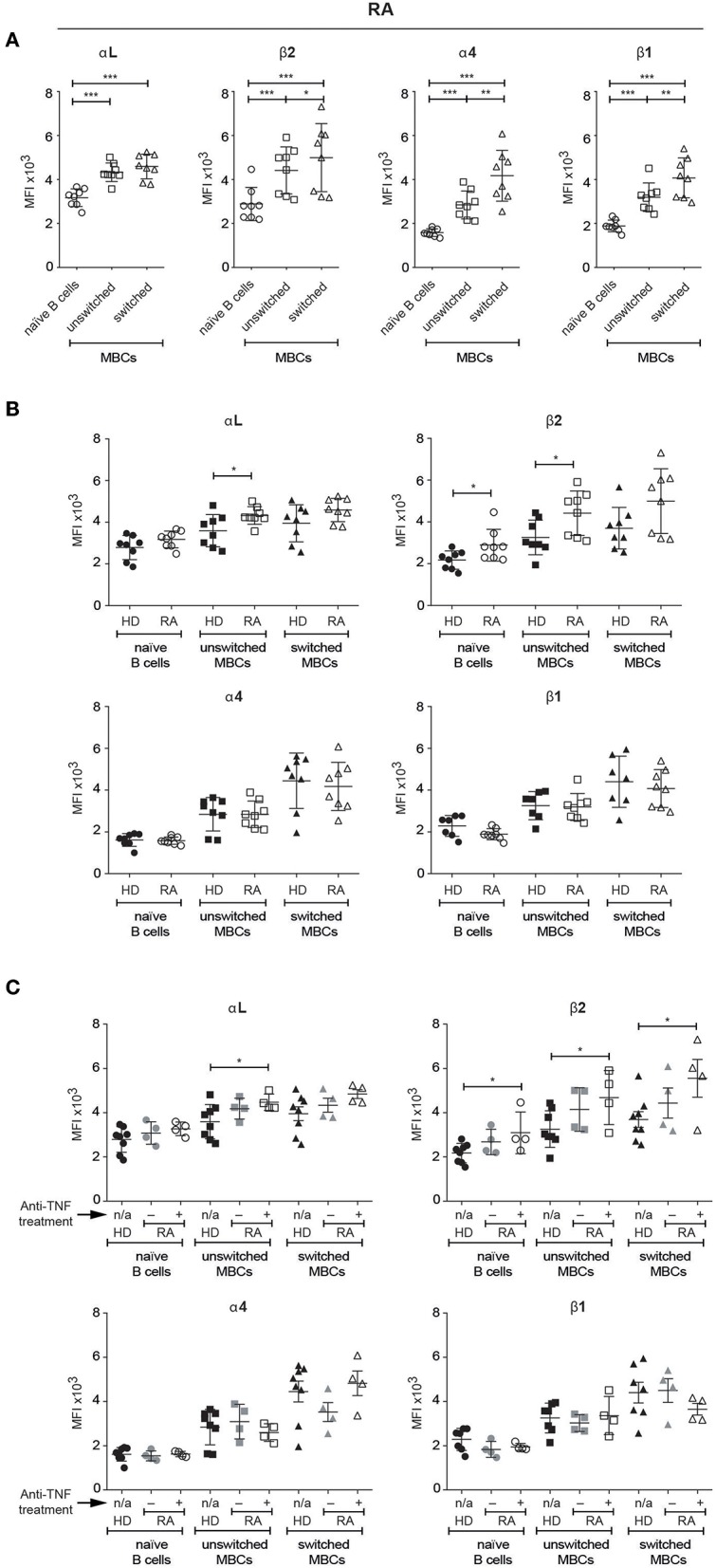
Human unswitched MBCs from patients with RA show an upregulation of LFA-1 subunits. Flow cytometric analysis comparing the expression of αL, β2, α4, and β1 integrin subunits amongst selected B cell populations in PBMCs **(A)** Patients with RA **(B)** Comparison between HDs and patients with RA treated with anti-TNF medication or not **(C)** Comparison between HDs and patients with RA with or w/o anti-TNF medication. Data are pooled from two independent experiments. Error bars show mean +/– SD. An unpaired two-tailed Student *t-*test was used (^*^p < 0.05, ^**^p < 0.01, ^***^p < 0.001).

To test our observations on autoimmune mice in humans, we chose to investigate patients with RA, following studies by others in which antibody blockade of ICAM-1 in such patients was reported to have beneficial effects on various clinical parameters, although no data on the effects on B cells were published (16). As for the MBCs from healthy donors, we found that the MBCs in peripheral blood from patients with RA expressed higher levels of all four integrin subunits of interest than the naïve B cells (Figure 6A). Additionally, and as observed in PBMCs and tonsils in healthy donors, both VLA-4 subunits (α4 and β1) appeared at significantly higher levels on switched than unswitched MBCs.

Comparing B cells in peripheral blood from healthy donors and patients with RA revealed some significant elevations of LFA-1 subunits expression in the RA samples: unswitched MBCs had higher levels of both αL and β2, and naïve B cells had a higher level of β2 than their counterparts from HDs (Figure 6B). As the patients appeared to split into two groups, we looked for an association between the number of swollen/tender joints and integrin expression, but found none (data not shown). In contrast however, dividing the patients into those that receive anti-TNF treatment and those that do not revealed significant differences in LFA-1 expression (Figure 6C). Those treated with anti-TNF had higher expression than HDs of the αL subunit on unswitched MBCs, and of the β2 subunit on all three B cell populations identified.

### The Expression of the Active Form of αL can be Induced by Pre-treatment With ICAM-1 and VCAM-1

**Figure 7:**
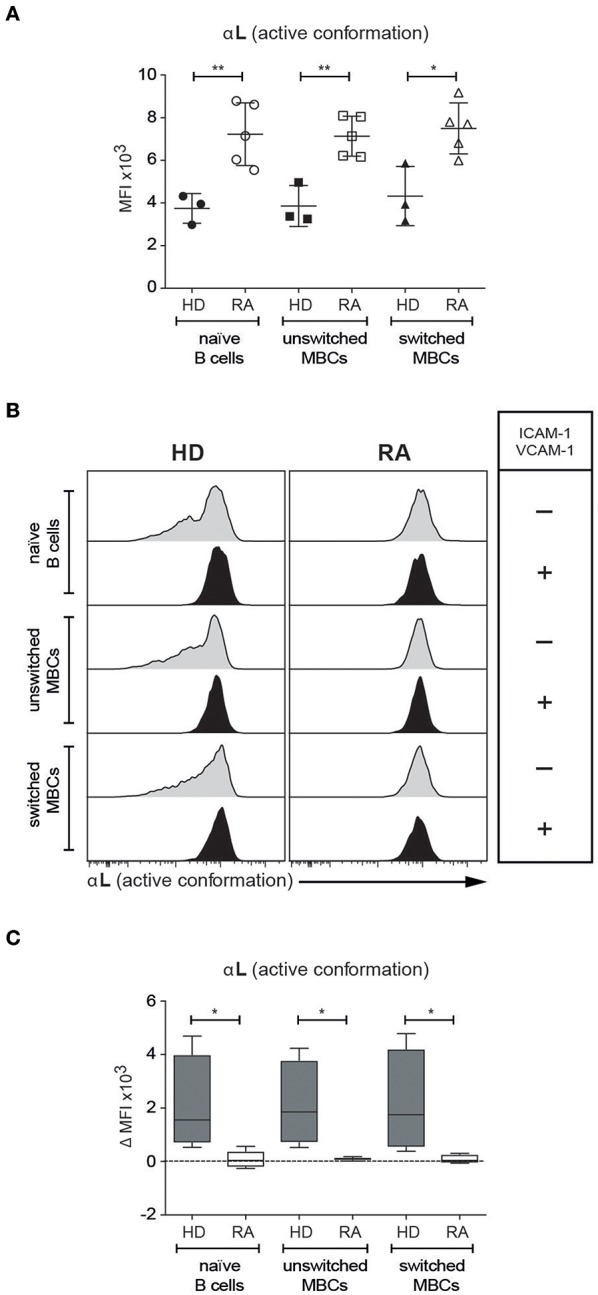
Human naïve, unswitched and switched MBCs from patients with RA show an upregulation of the active form of αL **(A)**. Comparison based on flow cytometric analysis of the expression of the active form of αL integrin subunit amongst selected B cell populations in PBMCs from HD and patients with RA under treatment with anti-TNF medication **(B,C)**. Staining for the active form of αL on PBMCs from HDs and patients with RA under treatment with anti-TNF medication, following pre-treatment with ICAM-1 and VCAM-1 **(B)** Expression of αL before and after treatment with ICAM-1 and VCAM-1 **(C)**. ΔMFI of αL due to treatment with ICAM-1 and VCAM-1. Data are pooled from two independent experiments. Error bars show mean +/- SD. Unpaired **(A)** or paired **(B,C)** two-tailed Student *t-*tests were used (^*^p < 0.05, ^**^p < 0.01).

The antibody that was used to stain for β2 integrin expression recognizes the active form of this subunit (17). We decided, therefore, to investigate also the expression levels of the active form of αL using an antibody known to recognize this form, selecting RA patients treated with anti-TNF medication. Our analysis showed that expression of the active form of αL was elevated relative to HDs on naïve, unswitched and switched MBCs from patients with RA, amongst which active αL expression was invariant across the B cell populations (Figure 7A). Thus, circulating naïve B cells as well as unswitched and switched MBCs in RA patients on anti-TNF medication express the active form of both subunits of the LFA-1 integrin.

It has previously been shown that patients with RA have higher levels of circulating ICAM-1 (18). To see whether the higher expression levels of the active form of αL seen in patients with RA could be induced in healthy donors in the presence of the ligand, we pre-treated PBMCs from both HDs and patients with ICAM-1 and VCAM-1. In consequence, the level of αL expression on naïve, unswitched and switched MBCs from HDs increased, matching those of their counterparts from the RA patient group (Figures 7B,C), which remained unaffected by the pre-treatment. Thus, the presence of ICAM-1 and VCAM-1 can trigger an increase in the activated form of αL expressed on the surface of PB B cells in healthy donors, to levels already expressed on the B cells of patients with RA.

## Discussion

Immunological memory depends on the retention of long-lived lymphocytes primed against specific antigens; as a consequence of this requirement, secure retention of memory cells in appropriate locations is essential. It has already been shown that VLA-4 and LFA-1 are crucial for the retention of MZ B cells in the murine spleen (2, 3). These molecules are therefore likely candidates for maintaining a functional MBC population in secondary lymphoid tissues, and as regulators of B cell trafficking. Here, we provide evidence that they are indeed responsible, via their enhanced expression on MBCs, for the selective retention of B cell memory at sites where ICAM-1 and VCAM-1 occur. Our blocking experiments demonstrate that both LFA-1 and VLA-4 have a role to play, but that their differential expression on MBCs relative to MZ B cells provides for selective placement of the subsets in lymphoid tissues, VLA-4 exerting the stronger pull for MBCs, and LFA-1 being the dominant influence on MZ B cells.

Unswitched human MBCs are known to be located in the splenic marginal zone, whereas switched MBCs are found in the perifollicular zone (19, 20); our results predict that a significant measure of their positional specificity is mediated by ligation to VCAM-1 expressed in these zones (4), and by inference also ICAM-1. For the unswitched MBCs, which depend on the spleen for their generation and survival (8, 21, 22), this receptor-ligand pairing may even be of developmental importance. The enhancement of adhesion capacity of both unswitched and switched MBCs over naïve B cells that we demonstrate is likely to reflect the differential importance for the two subsets of staying in one place.

B cells are one of the key players in RA, as is evident in the beneficial effects of B-cell depletion therapy (23). They are also prominent in a mouse model of RA, collagen-induced arthritis, in which the incidence of arthritis is substantially reduced in the absence of the LFA-1 ligand, ICAM-1 (24). It has been known for some time that ICAM-1 and VCAM-1 are expressed in the synovium and on chondrocytes of patients with RA (25–28), and for not quite so long that MBCs can be found in RA synovium (29). These previous observations are extended by our findings that levels of the active conformations of the LFA-1 subunits are elevated on the B cells of patients with RA on anti-TNF medication, and that the presence of ICAM-1 and VCAM-1 can induce similarly elevated levels of the active αL subunit on B cells from healthy donors. The implication is that the adhesion capacity of B cells, especially MBCs, may be a mediating factor in the development of sites of inflammation in RA. Our data would predict that the beneficial effects of blocking ICAM-1 in some patients with RA (16) might be due, at least in part, to the dual outcomes of reduction in the ICAM-1 induced elevation of active subunit expression, and prevention of MBCs expressing the active form of LFA-1 from adhering to the synovial tissue.

Integrins expressed on B cells, and in particular MBCs, have been found to be relevant to autoimmune conditions other than RA. In multiple sclerosis, interference with the integrin-mediated trafficking of leukocytes to the brain using a blocking antibody to the VLA-4 subunit α4 (natalizumab) results in B cells being trapped in the circulation (30). In systemic lupus erythematosus it has recently been shown that a MBC population expresses a transcriptional profile that includes increased levels of the adhesion molecules αL and β1 (31). Simple blocking of integrin molecules as a therapy for such autoimmune states carries the risk of side effects such as infection (32), and efforts are already being directed toward more specific inhibition; for example, inhibitors targeting gut-specific integrins are now under evaluation for Ulcerative Colitis (33).

Collectively, our results demonstrate that LFA-1 and VLA-4 are more highly expressed on MBCs in both mice and human than on the respective circulating naïve B cells, and MBCs in humans adhere better to ICAM-1 and VCAM-1 than do naïve B cells. Murine MBCs are retained in the spleen by both LFA-1 and VLA-4, but VLA-4 holds the dominant influence via higher expression levels. These data constitute significant steps toward the deciphering of the mechanisms behind lymphocyte retention, release, and trafficking. This understanding could reopen the use of a more specific integrin blockade to address several autoimmune diseases; blocking MBCs from entry into as well as forcing their release into the circulation from the spleen and inflammatory sites could break the positive feedback loop that nourishes autoimmune pathology.

## Data Availability

All datasets generated for this study are included in the manuscript and/or the supplementary files.

## Ethics Statement

This study was carried out in accordance with the recommendations of the regional ethical review board of Gothenburg and of the local ethical committe of Bambino Gesu Children's Hospital, Rome with written informed consent from all subjects. All subjects gave written informed consent in accordance with the Declaration of Helsinki. The protocol was approved by the regional ethical review board of Gothenburg and by the local ethical committee of Bambino Gesu Children's Hospital, Rome. This study was carried out in accordance with the recommendations of the regional animal ethics committee in Gothenburg, Sweden. The protocol was approved by the regional animal ethics committee in Gothenburg, Sweden.

## Author Contributions

AC, NG, OG, and I-LM designed the experiments. AC, CF, DC, IG, KT, LSO, NG, OG, RC, TF, and YW carried out or contributed essential reagents and materials for the experiments. AA and SC contributed substantially to the discussions. AC, I-LM, NG, and OG wrote the manuscript with contributions from the co-authors.

### Conflict of Interest Statement

The authors declare that the research was conducted in the absence of any commercial or financial relationships that could be construed as a potential conflict of interest.

## Abbreviations

Ab: antibody
FO: Follicular
HD: Healthy Donor
ICAM-1: Intercellular Adhesion Molecule 1
LFA-1: Lymphocyte function-associated antigen 1
MBC: Memory B Cell
MZ: Marginal Zone
PBMC: Peripheral Blood Mononuclear Cell
RA: Rheumatoid arthritis
SLC: Surrogate light chain
VCAM-1: Vascular cell adhesion protein 1
VLA-4: Very late antigen-4.
