# Supplementary material for: Dissecting Integrin Expression and Function on Memory B Cells in Mice and Humans in Autoimmunity
Source: Front Immunol. 2019 Mar 21;10:534. doi: 10.3389/fimmu.2019.00534 (PMC6437070; doi:10.3389/fimmu.2019.00534)
Supplement: Supplementary file 1 [file Data_Sheet_1.docx]

**Supplementary Material**

**Dissecting integrin expression and function on memory B cells in mice and humans in autoimmunity**

Alessandro Camponeschi^*^, Natalija Gerasimcik^*^, Ying Wang, Timothy Fredriksson, Dongfeng Chen, Chiara Farroni, Katrin Thorarinsdottir, Louise Sjökvist Ottsjö, Alaitz Aranburu, Susanna Cardell, Rita Carsetti, Inger Gjertsson, Inga-Lill Mårtensson^#^ and Ola Grimsholm

^#^Correspondence: Dr. Inga-Lill Mårtensson; [lill.martensson@rheuma.gu.se](mailto:lill.martensson@rheuma.gu.se)


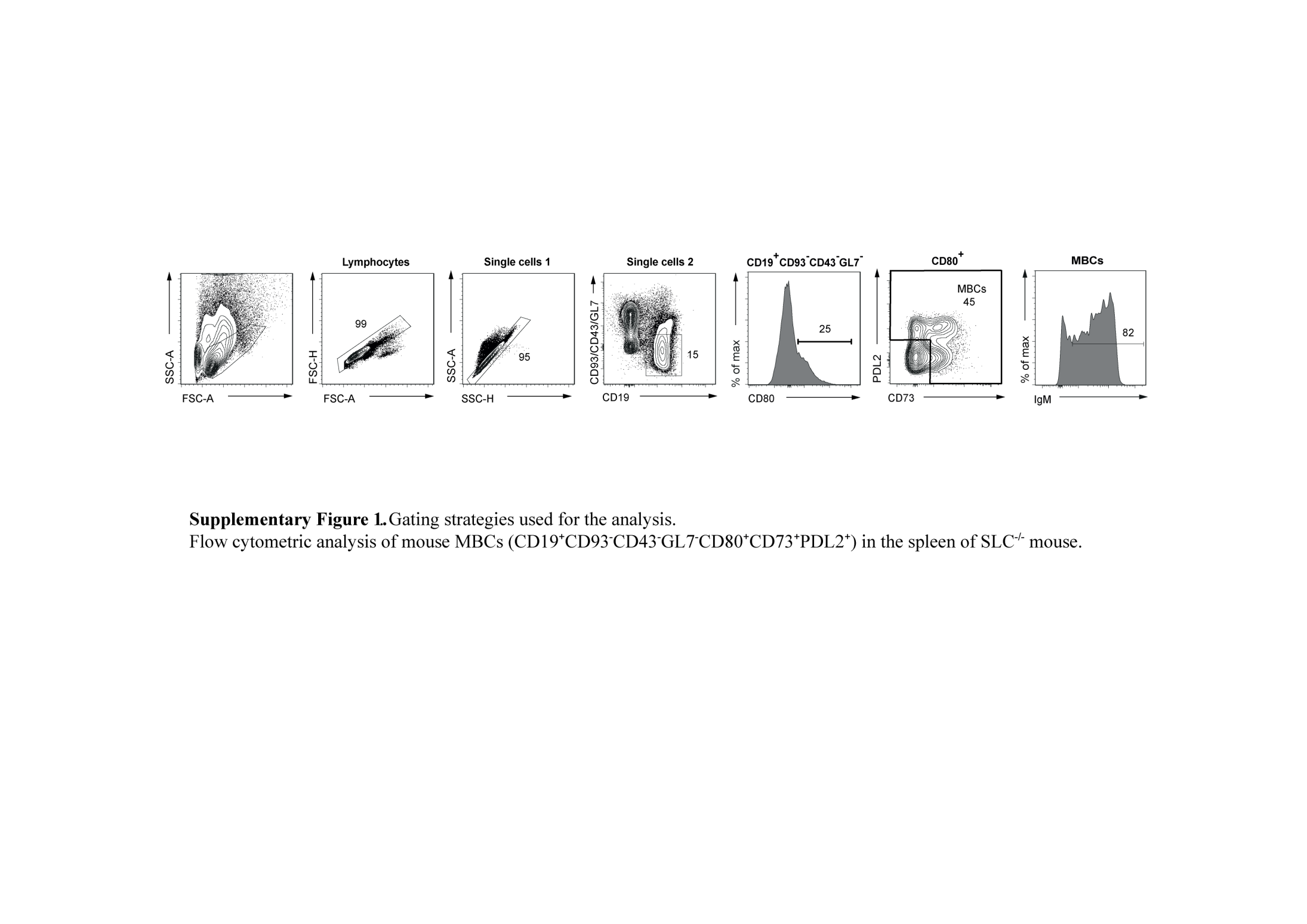


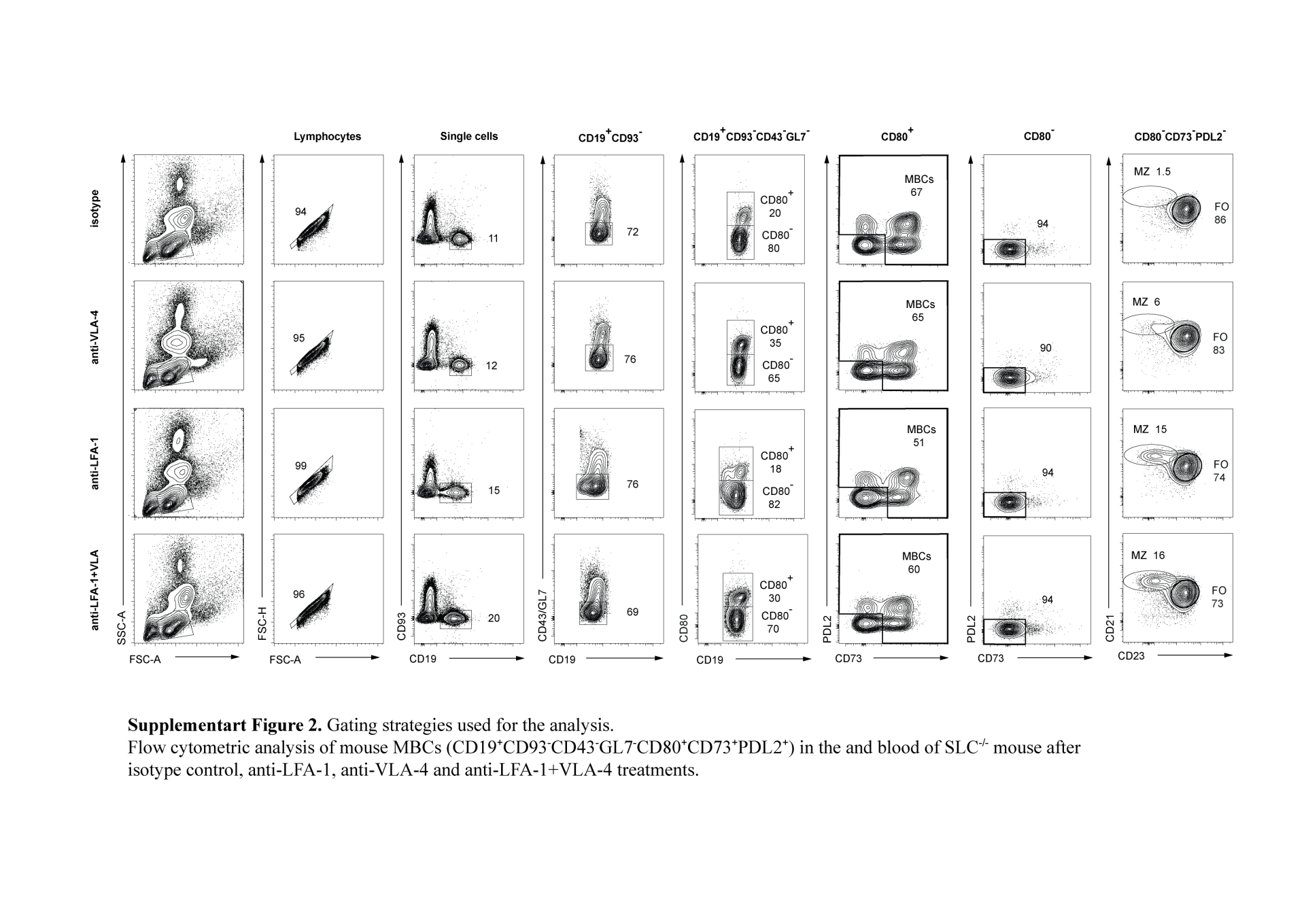


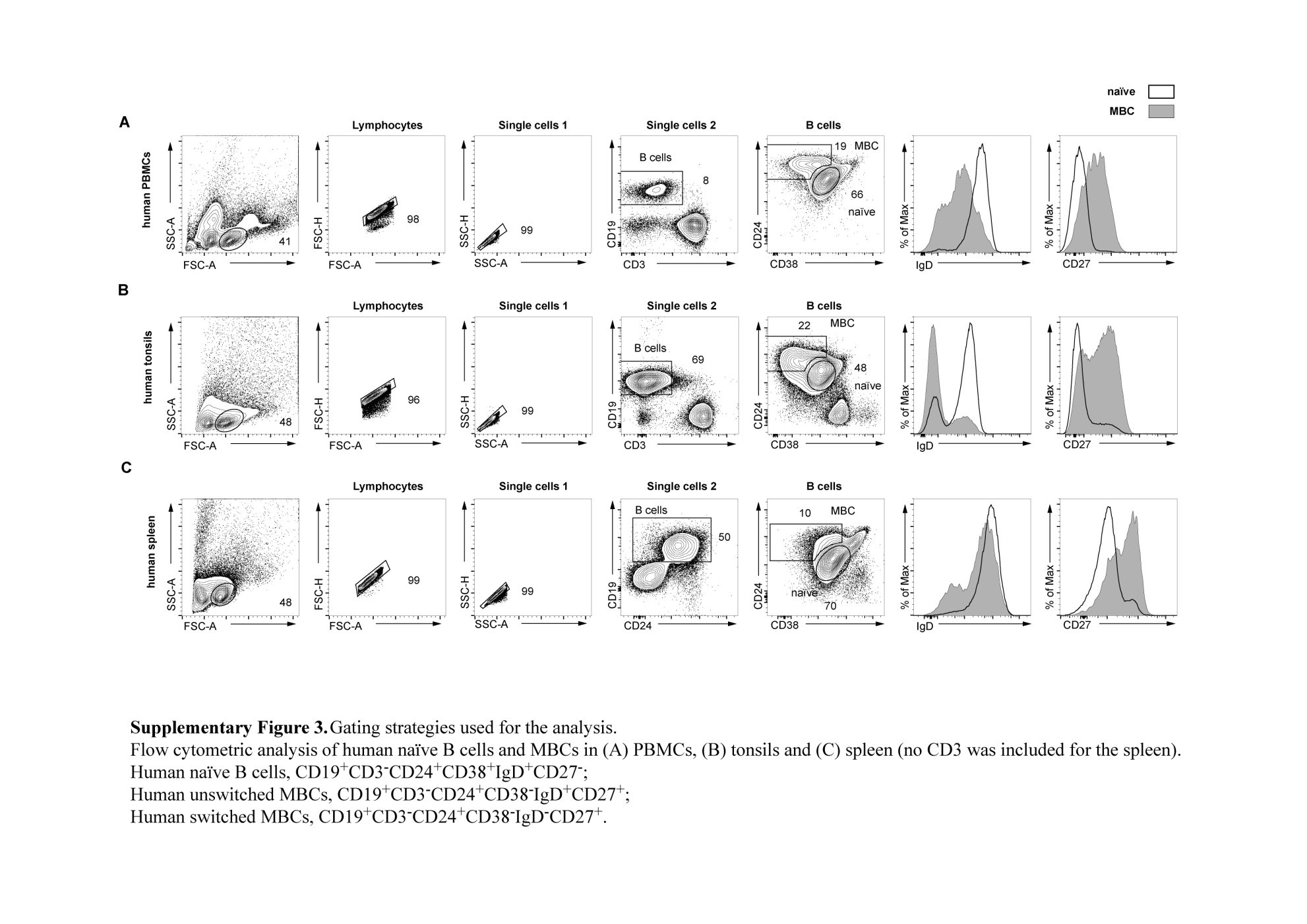

**Supplementary Table 1.** Clinical characteristics of patients with rheumatoid arthritis included in the study.

**Supplementary Table 2.** Anti-mouse antibodies used for the flow cytometry.

| **Antibody** | **Clone** | **Company** | **Dilution** |
| --- | --- | --- | --- |
| B220-BV605 | RA3-6B2 | BioLegend | 200 |
| B220-PerCP | RA3-6B2 | BD | 300 |
| CD11a-APC | M17/4 | BioLegend | 100 |
| CD11a-PE | 2D7 | BioLegend | 100 |
| CD16/32 | 2.4G2 | BD | 800 |
| CD18-AF647 | M18/2 | BioLegend | 50 |
| CD18-FITC | C71/16 | BD | 100 |
| CD19-V450 | 1D3 | BD | 200 |
| CD19-BV510 | 1D3 / 6D5 | BD / BioLegend | 400 |
| CD21-APC-Cy7 | 7E9 | BioLegend | 200 |
| CD23-BV510 | B3B4 | BioLegend | 200 |
| CD23-PerCP-Cy5.5 | B3B4 | BioLegend | 400 |
| CD29-APC | eBioHMb1-1 | eBioscience | 100 |
| CD43-APC | S7 | BD | 600 |
| CD49d-APC | R1-2 | BioLegend | 200 |
| CD73-PeCy7 | TY/11.8 | eBioscience | 400 |
| CD80-biotin | 16-10A1 | eBioscience | 800 |
| CD93-APC | AA4.1 | eBioscience | 800 |
| CD95-BB515 | Jo2 | BD | 200 |
| CD273(PDL2)-BV421 | TY25 | BD | 400 |
| GL7-AF647 | GL7 | BD | 300 |
| GL7-eFl450 | GL7 | eBioscience | 80 |
| GL7-FITC | GL7 | BD | 300 |
| IgM-AF488 | Polyclonal goat F (ab’)2 anti-mouse | Southern Biotech | 1000 |
| Streptavidin-PE | - | eBioscience | 1500 |
| Streptavidin-PeCy7 | - | BD | 600 |

**Supplementary Table 3.** Anti-human antibodies used in the study

| ***Antibody*** | ***Clone*** | ***Company*** | ***Dilution*** |
| --- | --- | --- | --- |
| CD3-APC-H7 | SK7 | BD | 50 |
| CD11a-PE | MEM-25 | Exbio | 20 |
| CD11a-purified | MEM-83 | ThermoFisher | 20 |
| CD18-PE | MEM-48 | Exbio | 20 |
| CD19-PE-Cy5 | HIB19 | BD | 20 |
| CD19-BV510 | HIB19 | Biolegend | 40 |
| CD24-PE-Cy7 | ML5 | BD | 40 |
| CD24-BV711 | ML5 | BD | 20 |
| CD27-BV605 | L128 | BD | 10 |
| CD27-PE | L128 | BD | 40 |
| CD38-BV421 | HIT-2 | BD | 20 |
| CD29-PE | MEM-101a | Exbio | 20 |
| CD49d-PE | 9F10 | Exbio | 20 |
| IgD-PE | IA6-2 | BD | 40 |
| IgD-FITC | IA6-2 | BD | 50 |
| IgM-AlexaFluor647 | 109-606-129 | Jackson Immunoresearch | 400 |
| Anti-mouse IgG1-PE (secondary antibody) | A85-1 | BD | 20 |
